# The legacy of care as reflexive learning

**DOI:** 10.1590/1518-8345.0639.2711

**Published:** 2016-06-14

**Authors:** Marta Rodríguez García, Jose Luis Medina Moya

**Affiliations:** 1 PhD, Adjunct Professor, Facultad de Biomédicas y de la Salud, Universidad Europea de Madrid, Madrid, Spain.; 2 PhD, Full Professor, Facultad de Pedagogía, Universidad de Barcelona, Barcelona, Spain.

**Keywords:** Education, Nursing, Preceptorship, Education, Higher, Learning, Clinical Clerkship, Concept Formation

## Abstract

**Objective:**

to analyze whether the tutor's use of reflexive strategies encourages the students to reflect. The goal is to discover what type of strategies can help to achieve this and how tutors and students behave in the practical context.

**Method:**

a qualitative and ethnographic focus was adopted. Twenty-seven students and 15 tutors from three health centers participated. The latter had received specific training on reflexive clinical tutoring. The analysis was developed through constant comparisons of the categories.

**Results:**

the results demonstrate that the tutors' use of reflexive strategies such as didactic questioning, didactic empathy and pedagogical silence contributes to encourage the students' reflection and significant learning.

**Conclusions:**

reflexive practice is key to tutors' training and students' learning.

## Introduction

Departing from the notion of reflexive practice^1^ ^-^ ^2^, which is considered a complex process that produces professional knowledge, with relevant intervening aspects like intuition, creativity and personal experiences^3^, it seems necessary to start and cultivate reflexive knowledge that makes professional action meaningful. In that sense, reflexive practice is a means to support innovative practice^4^ ^)^ that permits analyzing the capacity to reflect on the experiences lived^5^. In professional practice, we are still influenced by the premises of the positivist paradigm. The students imitate the professionals without any individual reflection process^6^. Reflexive practice is the main change. The intent is to develop a culture that values discovery and ongoing experimentation^5^. The key aspect is how to contribute for the students to capture the mystery of the care function and be capable of adapting it to the patient's concrete and individual situations and to the practical situation.

This refers to a new form of learning and a new role for the teachers, in a more student and experience-centered model^4^. Educational programs are confronted with the challenge of developing reflexive skills in the students with a view to providing better quality nursing care, favoring growth and professional development^7^. In nursing, rethinking practice requires the development of reflexive skills and the increased awareness of the complexity and diversity of care^8^.

Objective: to analyze whether the use of reflexive strategies contributes to encourage reflection in the students. 

## Method

Qualitative ethnographic design. Twenty-seven students participated from three Nursing colleges (seven men and 20 women, between 21 and 26 years of age), as well as 15 tutors from three health centers (two men and 13 women, between 27 and 48 years of age), who had received specific training on reflexive clinical tutoring. The data were obtained by triangulating different methods: non-participant observation of the educational practices and reflexive tutoring between tutors and students, in-depth interviews and discussion groups held in the course of three years by the main investigator (MRG), who was a faculty member of a nursing college and had 12 years of clinical experience. The courses took 30 hours, including a theoretical part, when the professionals tried out the Reflexive Tutoring Focus on Clinical Supervision and some of the reflexive strategies; and a practical part, when the tutors and students took part in reflexive conversations on a practical situation they had shared. Between the theoretical and practical part, a period for training was at the tutors' disposal. The tutors were selected based on availability of time and coincidence between their tutoring activities and the courses. The students had been assigned to their post. They were asked to participate voluntarily and received previous explanations about the study objectives. The relation with the participants was established during the course. The course coordinator at each center was available at all times. During the final course session, the preliminary results were presented to the tutors and students. 

In parallel, the observation periods took place at the different units and the in-depth interviews were held with the tutors and discussion groups with tutors and students. In total, 115 hours of non-participant observation were held, two individual interviews with tutors, two group interviews with tutors and four group interviews with students and 22 reflexive tutoring sessions were recorded between tutor and student. The length of the interviews and discussion groups was two hours at most. The data were registered in a video camera for further transcription. The tutors and students validated all data. The data collection was closed off when the saturation point was reached. To analyze the data, the constant comparison method proposed by Glaser and Strauss^9^ was used. The analysis starts with the search for the first-order concepts^10^, descriptions and interpretations the tutors and students developed with regard to the phenomena. The researcher developed the analysis. Sixty-eight first-order concepts emerged. At a second analysis level, eight thematic units or second-order concepts emerged: reflection as a source of learning, experience as a source of knowledge, tutor-student relationship, pedagogical activities, the art of the question, the role of the tutor, the role of the student and the legacy of professional identity. After the linear and cross-sectional analysis of the thematic units, the three theoretical constructs were developed which we call qualitative vectors and which subsumed the entire reality analyzed: the pedagogical relationship, experience-based learning and reflexive learning. In this study, a summary of the third vector is presented: reflexive learning. To facilitate the systematic and strict search and retrieval of the data, the software Atlas-ti version 6.2 was used. 

## Results

Next, the reflexive strategies observed are studied during the research are presented in the words of the protagonists. 

### Didactic questioning

In the course of the fieldwork, it could be observed how the tutors often dialogued with the students. Through the linkage of questions and answers, they incorporated the students' thinking in a dialectic process of reflection and learning. In the following example about care for an emergency patient diagnosed with myocardial infarction by the out-of-hospital emergency care team that had brought him to the hospital, through the questions, the tutors guides the learning by linking questions and answers, so that the student's thinking is driven towards the discovery of the ideas and/or procedures the tutor wants to show her. The student starts explaining the actions performed to maintain proper ventilation.

*So one of the nurses stands on the left side and installs the pulse oximeter to measure the saturation, showing that the patient has dyspnea and 9-% saturation. His glasses are changed and a 50% mask is placed (S).*

The tutor formulates a first question when she detects that, in her report, the student omits relevant aspects of nursing care: the patient's position*.*

*Before that, also, what would you do? A slightly simpler step than putting on a mask. Something that could make the patient feel much better than with a mask (T). Open his airway? (N)*

In view of that answer, the tutor immediately notices that the student did not perceive certain actions that were actually performed to enhance the patient's ventilation and thus asks those questions that could help her better to guide the student's thinking towards the desired learning. Thus, through a new link of questions and answers, she reorients the student's reflection in order to look tighter into the actual case as it happened. 

Let us depart from the baseline that the patient arrives with dyspnea, but breathing: permeable airway. *But you, when you choke, what do you do? (T).*

*Cough (N).*

*And what is your posture? (T).*

*I bend over (N).*

*You bend over. Exactly. We'd do the same with the patient. Put him in a position of 30-45º. That improves the breathing and reduces the patient's anxiety. Myocardial oxygen consumption always increases as a result of anxiety (T).*

As shown, in this type of dialogues, multiple questions can be asked and orientations can be provided which, as a form of clues, help the students built new knowledge. At all times, departing from the student's answers, the tutor introduces important contents and ideas about professional practice. Nevertheless, this episode also shows that reflexive tutoring always requires some degree of mental connection between tutor and student in the framework of a joint action, through which the tutors intentionally get into the students' mind to discover their perspectives, asking how they go to that idea or notion. Thanks to these "interpretations", the tutors are in tune with the students and "diagnose" their understanding of the content they are to learn *in situ*. 

### Reflexive tutoring: asking while teaching.

As reflexive professionals, the tutors need to master the art of the question and should be aware of the need to continuously encourage the students to put in practice their individual reflection processes, reflecting in and about the action. The tutors use four forms of questioning that give room to distinguished learning contexts/environments. 

2.1 Reflexive questions. These reflexive questions provoke the main possibilities of reflection in the students, as they oblige them to analyze their own thoughts on care. Given their enormous educational potential, these are the questions the tutor should use most. They emerge spontaneously upon the tutor's initiative and frequently mark the onset of the reflexive dialogue or Socratic conversations. In the students, they provoke moments of reflection in and about the action: 

*He did not have a cardiorespiratory arrest, he was breathing and had a pulse. Why do you think the cardiopulmonary reanimation cart was brought? (T).*

### Questions answering other questions

The tutor raises these questions to allow the students themselves to answer the questions they have just asked the tutor. They emerge upon the students' initiative and it is the tutor who transforms them into reflexive questions when using another reflexive strategy discussed further ahead: pedagogical silence. These questions imply the tutor's capacity to identify which lack of understanding lies at the base of the question and to imagine a new question, intentionally asked to get an answer that serves as a "bridge or cognitive threshold", whose crossing "brings the student closer" to the solution of his first questions. In the following dialogue, tutor and student reflect on the central venous tracts and the central venous pressure:

*This, what's this? (the student grabs one of the cables of the arterial system), if this leads to the artery, where does that one go? (S).*

*What are we measuring there? (T).*

Instead of giving a direct answer, the tutor starts a Socratic dialogue and does not answer the student, so that he finds the knowledge himself. She starts her line of reasoning by introducing increasingly complex questions with regard to what the student is answering. The student adopts a reflexive gesture. 

*I'll tell you, we are measuring a central venous pressure, where can you measure a central venous pressure? (T).*

*Where? (S).*

*Yes (T).*

*Well, directly from the central venous tract you have channeled (the student marks his neck) (S).*

*Through any central venous tract, through a pulmonary catheter, through any central catheter is good? (T).*

*Yes (S).*

*Exactly, the central venous pressure, what's that? What are you measuring? (T).*

*It's the pressure in the right atrium (S).*

*Hence, you'll have to put it in the lumen going to the right atrium, right? In a central venous tract which lumen goes to the right atrium? (T).*

*Ah yes, that's true, you mentioned that the other day in class (S).*

### Rhetorical questions

Are questions the tutor asks to develop her argument and to answer herself. These questions do not favor the other person's reflection, as they are part of the reflexive processes of the person asking them. They could be useful to maintain the attention on the actual discourse and arouse reflections in the person asking. The power of this type of questions lies in the capacity to generate reflection in the action at the heart of an argument, generally the tutor's, and sometimes the student's:

*If there's little blood volume arriving, there will be little venous pressure, if there's great blood volume arriving there will be a lot of pressure. So, what do you have to do to increase that venous pressure? Increase the volume, you see? (T).*

### "Rain" of questions

Consists in linking a series of reflexive questions, usually distinct forms of asking the same aspect of care. They tend to be used to help the student understand the question that is being asked. Although the initial effect they tend to produce in the student involves anxiety and confusion, when the tutor starts giving answers and shows the relations between them, she offers a good opportunity of "scaffolding" to allow the students to integrate distinct knowledge they had considered disconnected thus far. 

*Why do you need to measure the venous pressure. For people whose venous pressure is really low, what will you indicate? (T).*

As we have seen thus far, the tutors can develop reflexive strategies, such as didactic questioning and asking while teaching, because they have a set of schemes that help them to perceive the disorder, the lack of understanding or the need to explain some aspect of care in the dialogues with the students. Then, through the linking of questions and answers, the tutors incorporate the students' thinking in a dialectic process of reflection and learning, which extends and takes form in function of the sequences of questions and answers that come up. In this type of conversations, the tutor asks questions and gives orientations that help the students to solve a problem or gain an in-depth understanding of the topic that is being discussed.

### Didactic empathy and its effect on the student's practical learning.

Through didactic empathy, the tutors value the students' response positively, mainly because these questions show how the students understand the situation in question, that is, they show the tutors their hypothetical understanding*. With this strategy, the tutors make continuous efforts to put themselves in the students' place and value their answers or comments, from the students' perspective and never from their view as experts. The students' hypothetical understanding is the tutor's starting point to gradually introduce more complex concepts from practice. 

Let us look at an example of this process of "inclusion" in the following reflexive conversation fragment on the endotracheal intubation procedure and on the function of the endotracheal tube cuff.

*What would we do with the tube? (T).*

*Well, you'd have to verify the cuff (S).*

*That's right, the endotracheal tube cuff (T).*

*We'd have to prove that it wasn't punctured before. Ten centimeters of air was introduced. After checking the tube could be introduced (S).*

*This cuff, what's its use once installed? (T).*

*So that it doesn't escape, to fix the tube you installed (S).*

*I agree: What else? Do you remember? (T).*

*To contain any hemorrhage, right? (S).*

The student feels authorized to openly raise their doubts to the tutor about the endotracheal tube cuff and, although her two answers are not what the tutor had hoped for, she considers them important and again reformulates the question. 

*Yes, that's logical, if there's a hemorrhage, for example, with the tracheotomy tubes, often the trachea is strongly inflamed and the cuff helps to inhibit the hemorrhage. Yes, that's logical, really, but what else does it serve for? (T).*

The tutor was not seeking this answer from the student, but it is surprising how she tries to translate what the students are saying at all times to make it meaningful and to find the reason what they said that. The student keeps thinking but does not know what the tutor wants to say, she helps her. 

*You'd have to bend over an intubated patient a little, because he always has to be slightly bent. (The tutor has started a sketch on paper while explaining her reasoning to the student). If you don't inflate the cuff, what happens? (T).*

*Ah, the air is lost at the sides of the endotracheal tube. (The tutor continues drawing) (S).*

*If you inflate the cuff ... (S).*

*You've isolated everything (S).*

*That's right, thank you. And not only with gastric content, with oral secretions too (T).*

*Exactly, because the secretions were also aspired (S).*

*That is why washing the intubated patient's mouth was so important and aspiring these secretions and keeping that mouth somewhat clean, right? (T).*

*Yes, yes, yes (S).*

### Pedagogical silence and its effect on reflection

According to the tutors, this "learning to be silent" is not an easy task. Pedagogical silence provokes many moments of reflection, when the students find themselves "forced" to a deeper insight into certain aspects of the practices. Nevertheless, for the pedagogical silence to arouse reflection, the student needs to be granted time and a pedagogical relationship needs to exist with the tutor, based on confidence and mutual respect. One of the tutors explains why she uses silence. 

*In order not to solve their doubt, often I even start asking questions to guide them, but they are accustomed and there will never be someone at their back to give them clues, you can do that at first... (T)*

For the students, the search for answers supposes great efforts and allows this learning they are discovering to gain significance.

*The students ask questions because it is easier for you to answer them than for them to think (T).*

The tutor is no longer the person the student can ask questions to, but has turned into the person before whom the student can ask himself questions.

## Discussion 

One of the strengths of the research is that the COREQ guidelines were followed. The tutors of the clinical nursing practices use a range of reflexive strategies. Although there are many studies on reflexive practice, almost of them have been developed in the academic sphere instead of practice^11^, which explains the importance of this research. In the light of the results found, the students seem to need orientation and continuous feedback to help them on their reflexive journey^12^. The awareness needs to be raised in the future professionals that daily actions should be accompanied by thoughts and reflections that grant meaning to this practice, in order to serve to improve care^13^. It is revealed that the reflexive focus in clinical tutoring helps the students, who lack professional experience, to turn these thoughts and reflections into words, which will be conscious and intentional at first but, over the years of professional experience^6^ ^,^ ^14^. This is about learning things while thinking about what one is doing, so that each executed act is by itself a new lesson that helps to improve the action. Giving up routines and automatic actions seems to be the premise needed for the student to acquire the habit of reflection^15^. The dialogue between tutors and students manifests that the reflexive strategies encourage the students to elaborate their own processes of thought more consciously, so as to generate their own knowledge and achieve significant learning. Some studies on reflexive practice have called these dialogues: learning conversations" or "direct meetings with the other"^16^. In the practical context, the reflexive conversations represent one of the most powerful mechanisms of collaborative reflection on aspects of care, which should commonly happen in the students' practical training. These reflexive meetings can range from planned tutoring to informal meetings that come up in practice. They can emerge before care practice, during care or after the action. In addition, the reflections can derive from the analysis of the activity the student has performed, which would be more beneficial; by the tutor or another professional. The tutors' role as practical-reflexive professionals is fundamental. To help the students reflect, the tutors need to have turned into reflexive professionals themselves^17^ and have to receive the education needed to develop their work with proper quality^18^ ^-^ ^19^.

In tutoring, the relation established between tutor and student plays a basic role. The student needs to feel free and trusting to show the tutor how he is understanding a certain aspect or professional situation. It is fundamental that they do not feel inhibited to question themselves in front of the tutor and that they can make mistakes in the tutor's presence, without fear of being recriminated^20^. A pedagogical relationship needs to be established between both, based on dialogue and mutual trust^21^. As a reflexive professional, during clinical practice, the tutor should serve as a guide and the student should play a more active and more protagonist role^22^. 

Some of the study limitations were the determination of the practicum by the students' participation in the clinical training and by the tutors' job planning. Another limitation that should be considered was the professionals' attitude of mistrust and fear when they were observed in their professional activity. This reticence, however, can be minimized through background knowledge on the context, on the relations with the protagonists and the stay in the practicum context. Nevertheless, this was taken into account at the start of the observation periods at the services and efforts were made to establish rapport with the protagonists as early as possible. 

## Conclusions

The study results, it is evidenced that tutors have a range of strategies at their disposal which they use with their students during clinical practice and which favor reflexive practice. It is also noticeable that the students' practical learning is produced at the heart of a pedagogical relationship, in which the student needs to stay with their clinical practice tutor continuously for the sake of a relationship of trust and freedom.

Both student and tutor should incorporate reflection as a habit in their daily practice. The reflexive conversations between tutor and student represent one of the most valuable mechanisms for the sake of reflection on the essential aspects of care. The tutors need to be able to use their reflexive skills in order to guide the student to understand the complex phenomena of practice. Therefore, it is important for the tutors to be educated and trained on how to use the reflexive strategies. In these reflexive encounters, the tutors will use didactic questioning, ask different types of questions and be empathetic by using silence. Through didactic empathy, the tutor needs to be capable of putting himself in the students' place, understanding their processes of thinking. In addition, they need to be aware that the use of silence is of pedagogical value to the students. The students are responsible for finding their own answers to the dilemmas of practice, taking an active role in their learning process, and the tutor will serve as a guide who accompanies the student, without imposing his judgment and watching over the student's autonomy. Reflexive practice represents a fundamental legacy in the education of future nursing professionals.

This study contributes to a further understanding of the learning process that takes places during the nursing students' clinical practice. Through reflexive practice, the training of practicum tutors improves and the students' significant learning is guaranteed. 
